# Enduring psychological impact of childhood cancer on survivors and their families in Ireland: A national qualitative study

**DOI:** 10.1111/ecc.13257

**Published:** 2020-06-15

**Authors:** Peter M. Barrett, Louise Mullen, Triona McCarthy

**Affiliations:** ^1^ HSE National Cancer Control Programme Dublin 1 Ireland; ^2^ School of Public Health, Western Gateway Building University College Cork Cork Ireland; ^3^ Wellcome Trust/HRB Irish Clinical Academic Training (ICAT) Programme University College Cork Cork Ireland

**Keywords:** cancer survivors, Ireland, oncology, psychological resilience, psychological trauma, survivorship

## Abstract

**Objective:**

To establish the major expressed psychological needs of adult survivors of childhood cancer living in Ireland.

**Methods:**

Seven focus groups were conducted with adult survivors of childhood cancer and their parents in 2018. Survivors were invited to participate if they were diagnosed with cancer before age 18.

**Results:**

Thirty‐three participants (15 survivors, 18 parents; 27 female, 6 male) were included. They had experienced a range of haematological and solid tumours. Five themes were generated: (a) Enduring psychological impact on survivors; many survivors experience delayed trauma and mental health crises in adulthood. (b) Enduring psychological impact on family members; parents and siblings have unmet psychological needs relating to the family's experience of cancer. (c) Enduring impact on family dynamics; survivors and parents expressed fear and guilt relating to cancer which impacted on family interactions. (d) Challenges accessing support; psychological support services are inadequate to meet expressed needs. (e) Desired model of care; no single service model appeals to all survivors, and flexibility is required in the delivery of psychological support.

**Conclusion:**

Adult survivors of childhood cancer and their family members experience enduring psychological effects relating to their diagnosis and treatment. Psychological support services are inadequate to meet the expressed needs of this growing population.

## 1 INTRODUCTION

Treatment outcomes for childhood cancer have consistently improved over recent decades, and the overall 5‐year survival rate for childhood cancer in Ireland is now over 80% (National Cancer Strategy 2017–2026, 2017). Although many survivors of childhood cancer enjoy good physical health as adults, psychological distress is common for a wide array of reasons (Brinkman et al., 2016). Some survivors face a range of medical challenges relating to late treatment effects. They are at heightened risk of complications such as recurrence of their primary cancer, secondary cancers, cardiac disease, infertility, ototoxicity or bone disease (Clemens et al., 2016; den Hoed et al., 2015; Gibson et al., 2018; Khan et al., 2018; Taylor et al., 2009; Wasilewski‐Masker et al., 2008). This can result in frequent ongoing contact with the health service and repeated hospitalisations (Brewster et al., 2014; de Fine Licht et al., 2017).

Some survivors experience anxiety relating to their health many years after completing their active cancer treatment, and adult survivors experience significantly higher levels of mental health dysfunction compared with the general population (Fidler et al., 2015). Large‐scale longitudinal studies have demonstrated higher rates of hospitalisation for mental disorders among survivors of childhood cancer (Lund et al., 2013), and higher prescription rates for anti‐depressants in this group (Deyell et al., 2013; Johannsdottir et al., 2017; Lund et al., 2015).

Some survivors experience profound social, cognitive and behavioural challenges at a vulnerable time in their development. A considerable minority report peer‐related difficulties and social isolation upon return to school, and this is particularly common among survivors of brain and central nervous system (CNS) tumours (Salley et al., 2014; Schulte et al., 2018). Survivors are more likely to underperform academically and again, survivors of CNS tumours are more susceptible due to poorer neurocognitive function (Barrera, Shaw, Speechley, Maunsell, & Pogany, 2005; Park et al., 2018; Zeltzer et al., 2009). Other vulnerable groups include those who are treated with cranial irradiation for other reasons (e.g. leukaemia) and those who are younger at cancer diagnosis (Barrera et al., 2005; Lancashire et al., 2010). Early cognitive and social difficulties can be perpetuated for some survivors, as they can lead to curtailed access to third level education, reduced employment opportunities, and financial hardship in adulthood (Gurney et al., 2009; Nathan, Henderson, Kirchhoff, Park, & Yabroff, 2018; Yabroff et al., 2016).

Previous research suggests that females, those with lower household income, and CNS tumour survivors are at highest risk of ongoing psychological distress (Zebrack et al., 2007; Zeltzer et al., 2009), but it is clear that other survivors of childhood cancer can be impacted too. Long‐term psychological impacts of cancer are likely to depend on the developmental stage of the child at diagnosis (Brand, Wolfe, & Samsel, 2017). Although generally attributed to the injurious effects of treatment, it has been argued that the adverse cognitive and behavioural sequelae may arise from altered neural development as a result of the “double hit” of an early threat to life and enduring invasive medical procedures during childhood (Marusak et al., 2018).

There is a dearth of data on the psychological impact of childhood cancer on adult survivors in Ireland, and this has been recognised as a growing problem in Ireland's National Cancer Strategy (National Cancer Strategy 2017–2026, 2017). We undertook a qualitative study to explore the perceived and expressed needs of this population, and to identify key priorities for adult survivors of childhood cancer, diagnosed before age 18. Given that childhood cancer is a relatively rare diagnosis in Ireland (Barrett, Mullen, & McCarthy, 2019), we included survivors of any form of early childhood or adolescent cancer, and parents of survivors, to get as broad an overview as possible of the major expressed needs of this group, and the expressed preferences for psychological support throughout survivorship.

## 2 METHODS

### 2.1 Study design

Adults were invited to participate in focus groups if they were diagnosed with any form of cancer before age 18, and were no longer under active treatment for cancer. Parents of children who had cancer within the past 20 years, and who were no longer under active treatment, were also invited to take part. A focus group methodology was chosen as this study design can provide detailed information on an area where there are insufficient prior data or knowledge (Liben, 2012). The focus groups involved wide‐ranging discussions related to perceived physical, psychological and social needs of adult survivors of childhood or adolescent cancer. In this study, we present the findings related to psychological needs.

Seven separate focus groups were conducted over a three‐week period in April 2018 (FG1‐7), and each focus group comprised of survivors only, or parents of survivors only. Focus groups were conducted in Dublin, Cork and Galway, and were scheduled to occur on a mixture of weekdays and weekends, during working hours and evenings to accommodate a diverse range of participants. The maximum number of participants in any focus group was eight.

Each focus group was facilitated by a trained researcher from the National Cancer Control Programme (NCCP) (PMB or LM) and a scribe was present to take notes. Participants were first asked to write down any thoughts or ideas they had regarding the major needs of survivors of childhood cancer in Ireland. After five minutes, each participant was asked to feedback individually to the group. After each participant had the opportunity to speak, two or three common topics were chosen for more in‐depth discussion between all participants. These topics were agreed by consensus between the facilitators, scribe and focus group participants. Topics were selected according to what issues arose most frequently during individual feedback, and appeared to resonate most with participants. In the latter focus groups, the facilitators encouraged discussion of topics which had not already been discussed or explored in detail in any previous focus groups.

An open‐ended topic guide was developed to elicit free‐flowing discussions between participants. Where possible, prompts were used to encourage elaboration on how gaps or deficiencies in services might be addressed or improved. After each focus group, a debriefing process took place between the interviewer and scribe to identify areas where more details were required, to reflect on the predominant emotions expressed by participants, and to modify the topic guide as needed.

### 2.2 Study promotion

The study was widely promoted by NCCP, Health Services Executive (HSE) Communications Division and by voluntary organisations all around Ireland. It was frequently advertised through social media on the Twitter accounts of HSE (>28,000 followers), NCCP (>800 followers), Irish Cancer Society (>38,000 followers) and several other voluntary organisations. Email alerts were sent out to 45 voluntary organisations around Ireland working with cancer survivors. These included organisations with wide areas of remit, including broad‐ranging cancer support (both general and tumour‐specific services), patient education, financial aid, psychological assistance, and peer support for affected individuals and family members. Individual meetings were held between NCCP and three of the leading voluntary organisations working in the area of childhood cancer to encourage promotion of the study. Professional networks of medical oncologists, haematologists, paediatricians and specialist nurses were informed of the study, and encouraged to invite participants.

A broadcast email was sent by the HSE Communications Division to all HSE staff members nationally to alert the wider public of the study, and to encourage participation. The study was also promoted in a national broadsheet newspaper and through local radio stations. Fifty expressions of interest were received to participate. Any participant who registered their interest and was available to participate in one of the seven focus groups was invited to take part. Where possible, participants were contacted by the lead author in advance of each focus group to ensure they understood the purpose of the study and its voluntary nature. Seventeen participants were unable to attend the focus groups at the times or locations available (7 survivors, 10 parents).

### 2.3 Ethical considerations

Ethical approval was obtained from the Clinical Research Ethics Committee of the Cork Teaching Hospitals in Ireland in March 2018 (Reference ECM4 07/03/18). At the beginning of each focus group, participants gave written informed consent to participate, with both the focus group facilitator (PMB or LM) and a scribe present. The consent forms emphasised the confidential nature of the information to be collected, and the right of participants to withdraw at any time.

In order to mitigate risk relating to discussion of psychological distress, we avoided asking direct, personal questions in relation to treatment. A separate, quiet room was available for participants if they became upset. Participants who experienced distress were signposted towards appropriate clinical caregivers (e.g. GP, psychologist), and all focus groups were facilitated by personnel with medical or psychology experience. Given that childhood cancer is a relatively uncommon diagnosis in Ireland, we did not seek any personal details relating to age (current, or at diagnosis), specific tumour type, or any other potentially identifiable information. We only present information which was disclosed voluntarily by participants.

### 2.4 Analysis

Focus groups lasted between 35 and 120 min and were audio‐recorded. The recordings were transcribed verbatim by an independent company. The transcripts were analysed using Braun & Clarke's Framework for Thematic Analysis to identify semantic and latent themes (Braun & Clarke, 2006). An inductive approach was used for analysis. Transcripts were first read repeatedly to gain an overall familiarity with the data, and early impressions were noted. Open coding was used to develop and modify initial codes. Two researchers (PMB and LM) undertook manual line‐by‐line coding of the first three transcripts independently as a means of fostering reflexivity, and these were reviewed and compared between the two researchers for consistency and face validity. The remaining transcripts were then coded by the lead researcher (PMB). Related codes were grouped together to form categories. These categories were then continuously reviewed to identify and generate themes and subthemes. Two members of the study team (LM and TMcC) audited the codes, categories, themes and subthemes to ensure they were objectively interpreted and to minimise potential for bias. This iterative process was repeated until there was consensus with the generated themes.

Sharing qualitative research findings with participants can enhance credibility of the findings and trustworthiness (Goldblatt, Karnieli‐Miller, & Neumann, 2011). Member‐check was undertaken with participants after the study team had interpreted the findings, and completed a preliminary analysis. Study participants were invited to two separate meetings, and the preliminary results were presented to them, and openly discussed in group settings. Participants were invited to comment on whether the preliminary findings truly reflected their insights, feelings and experiences. Informant feedback confirmed that the summaries reflected an accurate representation of their views and experiences.

## 3 RESULTS

In total, there were 33 participants: 15 survivors and 18 parents. The majority of participants (*n* = 27) were female. Of the six male participants, four were survivors and two were fathers of survivors. Survivors had experienced a diverse range of diseases, including leukaemia, lymphoma, brain/CNS tumours and osteosarcoma. The majority of survivors were currently aged 18–32 years, and they came from at least eight different counties in the Republic of Ireland. Their age at time of diagnosis varied widely, from early childhood (<5 years) to late adolescence.

Seventeen individuals who expressed interest in the study (seven survivors, 10 parents) were either unable to attend any of the focus groups (*n* = 14) or opted not to attend on the day (*n* = 3).

Five key themes were generated from the analysis.

### 3.1 Enduring psychological impact on survivors

Survivors in each focus group articulated how their cancer diagnosis and treatment had a negative impact on their overall wellbeing. Some experienced distress from an early age, but had difficulty verbalising this. Others felt that they had been resilient as children or adolescents, and that they were able to get through treatment without focusing on feeling upset. They described how they “blocked out” the emotional trauma, or hid their feelings in front of others. Some had difficulty processing their feelings until they were several years older.

Although narratives were individual, for those who had experienced psychological difficulties, most had not anticipated this occurring after completion of their treatment. For some, the long‐term effects of cancer treatment were described as “life‐changing,” and each new unexpected medical complication was “another trauma.” Several participants expressed regret that they had not been warned in advance of the potential for long‐term psychological impact. This hit them at a later age “like a ton of bricks,” often many years after completing treatment. A small number of participants described serious mental health effects which they attributed in part, or in full, to their diagnosis or treatment. One survivor described his need for crisis intervention for suicidality; another survivor suffered a “mental breakdown” and dropped out of university; and one parent described how her child had been hospitalised with repeated self‐harm and attempted suicide after completing treatment.

I ended up getting worse and worse, I went to (a suicide intervention agency) when it got to an intervention stage—Male survivor, FG1

Parents expressed concern that the psychological impact of treatment on younger children was often underestimated by healthcare professionals, and by the wider health service. One mother described how her 5‐year‐old son had planned his own funeral during treatment, and another mother described how her 2‐year‐old's distress manifested in his early speech development.

…the public health nurse wasn’t very pleased with the amount of words he had, but he learnt to speak very clearly within about two weeks (of the diagnosis) – ‘not the bloods, no more needles, I will be good, no more needles’—Mother of survivor, FG2

### 3.2 Enduring psychological impact on family members

The perceived need for psychological support for family members was repeatedly articulated by survivors and their parents. They were concerned about the wellbeing of siblings who were “pushed to one side” and “left to fend for themselves” during the treatment phase. Parents described the need for psychological support options for these “shadow children,” so that they could better process the upheaval that came with a sibling's diagnosis of childhood cancer.

Are they thinking their young brother or sister is going to die? Yes, of course they are. So who is looking after them? Nobody at the moment—Mother of survivor, FG2

Some parents described how these children had been “forgotten” or “abandoned” during treatment, and that some of these siblings had difficulty processing their own delayed trauma related to this time. They felt that parents should be advised on how best to support them throughout treatment and beyond.

…her older brother is 22 and he recently started counselling and it is directly linked to (his sister) being sick you know so he is still carrying that with him… he was eight when (she) was diagnosed first and… he is still carrying it—Mother of survivor, FG2

Parents themselves described high levels of distress during their child's diagnosis and treatment, but they felt particularly vulnerable at the end of treatment. This was described as the first time that they had a chance to reflect and try to process what had happened to them and their family. Many described this time as an important milestone which they had keenly anticipated, but which was overshadowed by mixed emotions. For some, instead of feeling joy and relief, they described it as an “anti‐climax” because it was their first opportunity to acknowledge their own personal anguish over the cancer diagnosis and treatment.

Several parents had assumed a carer role during the treatment phase, and in some cases had given up employment to do so. This added to the challenge of needing to readjust back to “normal life,” and they expressed a sense of loss over the life that they had given up. It was perceived that there was “nobody there” to support parents during or after treatment, and that the end of treatment was the first time that they could think about focusing on their own wellbeing, rather than on that of other family members.

A small number of parents spoke about personal experiences of mental health crisis. This was sometimes attributed in part, or in full, to the lack of psychological support available. One mother described locking herself in a hospital toilet during her child's treatment, and she required psychological intervention; another parent described having a “breakdown” after her child's treatment had finished.

… when you get go‐ahead that things are going to be okay that is often when the parent collapses and she falls down ill and falls apart because it is a very very lonely road as a parent—Mother of survivor, FG5

### 3.3 Enduring impact on family dynamics

Several parents identified themselves as “helicopter parents” or “mumcologists,” and they began to assume the role of nurse, physiotherapist or carer, as well as parent, during the treatment phase. Some participants described a very deep parent/child relationship during this time, where they spent almost all their time together and where the parent acted as the child's confidant. However, during follow‐up, some survivors expressed reluctance to divulge all information to their parents, particularly when it related to psychological distress. This was motivated by an underlying desire to take on greater levels of independence in their care, and a sense of guilt over the anxiety experienced by family members relating to their illness.

I just sometimes think I am a burden and I know I shouldn’t but I do with everything that has happened—Female survivor, FG6

Some parents struggled to withdraw from their children's care and remained highly vigilant for symptoms of recurrence or late effects during follow‐up, yet at the same time, they did not want to “smother their child.” One mother described her sense that “you just can't withdraw” around the time of transition from paediatric to adult follow‐up services, when the young person is still very vulnerable, and expressed guilt that this might lead to encroachment of her child's independence. There were multiple other expressions of guilt from both survivors and parents during the focus groups (Table 1).

**Table 1:** Summary of reasons underlying participants’ expression of guilt

Reasons underlying participants’ expression of guilt
Survivors’ guilt
Upset of family members during diagnosis and treatment
Continued upset of family members during follow‐up, particularly related to psychological effects and need to return to treating hospital
Financial burden imposed on family
Diversion of attention away from other siblings
Parental guilt
Lack of earlier recognition of child's illness, or lack of intervention at an earlier stage
Struggle to balance vigilance for symptom recurrence with desire to avoid encroaching on child's independence
Diversion of attention away from other family members
Seeking limited resources for their child when other families affected by childhood cancer may be worse off

Parents expressed a diverse range of fears which may further explain their reluctance to withdraw from follow‐up care. These included the initial fear for their child's survival; fear of recurrence of cancer; fear that they might miss an early sign or symptom; fear over a lack of access to services or supports; fear relating to their child's ability to readjust socially; and fear relating to the late effects of treatment. They described the fear of recurrence as persistent, and a source of distraction, “always at the back of your head.”

### 3.4 Challenges accessing psychological support

Participants described multiple challenges in accessing psychological support, either during or after cancer treatment. They were not always offered support at the time of diagnosis. Those who had received support during initial hospitalisation spoke positively about this. However, this support was typically transient, with little or no community‐based follow‐up once treatment had ended.

Survivors felt that formal psychological support should be offered as a default to all patients at diagnosis, and that the offer should be renewed throughout treatment and follow‐up. Survivors who attended medical appointments in the follow‐up period were rarely asked specifically about their psychological wellbeing. They were not inclined to volunteer this information unless directly asked. Some expressed disappointment that they were not routinely asked how they were coping with their illness. Furthermore, some survivors expressed difficulty articulating their emotions, or found it hard to open up to family and friends.

…they say it at home as well that I badly need to open up… I will cry at home to myself, I just get in a bad mood and I just go in to my room and I go for a nap or whatever, I don’t know what it is—Female survivor, FG6

Participants emphasised that there were major gaps in services, particularly in the public system. Those who were able to avail of free, formal psychological support in the public system described the services as “gold dust.”

Parents expressed frustration that they too could not routinely access psychological support. They articulated a desire for enhanced support services for entire family units from the time of diagnosis.

### 3.5 Desired model of care

Survivors were prompted on the essential components of the optimal model of care for improved psychological support. They described a service which was friendly, age‐appropriate, and which used accessible, non‐medical language. They expressed preference for a service which was entirely community‐based for those who finished medical treatment, and one which was relatively local to service users. There was no consensus on whether support should be offered on an individual or group basis, suggesting that some flexibility in service delivery would be optimal. Several survivors expressed preference for one‐to‐one support sessions, whereas others preferred small group sessions, and would be reluctant to engage in one‐to‐one counselling. Most felt that sessions should be facilitated by a psychologist, and a few participants specifically articulated the need for a psycho‐oncologist. Some parents of children with brain and CNS tumours emphasised the need for more neuro‐psychology support in the public system. A minority of participants felt their needs could be met through support sessions facilitated by non‐specialists, as long as they were professional, independent and empathetic.

For some participants, informal peer support was perceived to be a very valuable alternative to formal psychological services. Teenage support groups had provided some individuals with a therapeutic safe space, where they could conform with their peers again rather than feeling like “the odd one out.”

…getting to see (other teenagers) who I perceived as normal who had gone through it was enough for me and that was the first time I was optimistic about the whole lot—Male survivor, FG4

Some challenges to the provision of psychological support were raised. A minority of survivors expressed their own reluctance to speak about personal issues, and that they would be unwilling to engage in either one‐to‐one or group sessions. One survivor stated that she did not want to “dwell on the past,” but rather wanted to “get away from it” now.

I think I was just more keen on getting back to my normal life, getting back to what I knew best, so for me I felt I had no interest… I really didn’t want that (psychological support) at all—Female survivor, FG7

## 4 DISCUSSION

Survivors of childhood cancer articulated a strong need for improved psychological support during treatment and follow‐up in Ireland. There was no single service model suggested which would suit all survivors of childhood cancer equally, but the predominant message from survivors and parents was that there are major gaps in psychological supports in the community. Psycho‐oncology resources are scarce, particularly through the public healthcare system, and many families have no alternative but to access psychological support through voluntary organisations or by paying privately. There was consensus among survivors around the endurance of psychological distress following treatment, and that there is a need for a systematic approach to psychological assessment and care. This is consistent with international standards of care which recommend systematic assessment of psychosocial healthcare needs for childhood cancer survivors, and ready access to support and care throughout the cancer trajectory (Lown, Phillips, Schwartz, Rosenberg, & Jones, 2015; Wiener, Kazak, Noll, Patenaude, and Kupst 2015).

Delayed trauma was commonly experienced by survivors several years after finishing treatment. For some, this culminated in a serious mental health crisis requiring intervention. This is consistent with previous research from the UK and USA which identified higher levels of mental health difficulties and psychological distress among childhood cancer survivors compared with the general population (Fidler et al., 2015; Zeltzer et al., 2009), and higher levels of mental health service utilisation among survivors in Denmark (Lund et al., 2013). In Ireland, survivors are not routinely asked about their overall well‐being during medical follow‐up appointments. Although international guidelines recommend follow‐up screening for psychological, social and behavioural difficulties among survivors of childhood cancer (Children’s Oncology Group, 2018), this does not appear to be standard practice in the Irish context. Participants’ preference was that all survivors should be offered psychological support by default during treatment and follow‐up.

Although most participants in our study were concerned about their mental wellbeing and were fearful about their health, this did not apply to all. It is plausible that some survivors experience “posttraumatic growth,” or resilience that develops in coping with their adverse circumstances (de Laage et al., 2016; Mackie, Hill, Kondryn, & McNally, 2000). In cross‐sectional studies conducted elsewhere, high levels of psychosocial resilience have been reported among some survivors (Ford et al., 2015; Harila, Salo, Lanning, Vilkkumaa, & Harila‐Saari, 2010; Honda et al., 2011; Zeltzer et al., 2008). This has been positively associated with the presence of strong social support at the time of treatment and recovery, and negatively associated with long‐term physical effects (Yi, Zebrack, Kim, & Cousino, 2015). Further research is needed to improve understanding of survivors’ individual susceptibility to distress and mental health difficulties.

Our study suggests an unmet need for psychological support among family members. Some parents articulated enduring distress related to their children's cancer journey, and a minority experienced mental health crises. Posttraumatic symptoms are commonly experienced by parents of children receiving active treatment for cancer (Kearney, Salley, & Muriel, 2015). Parental distress usually decreases with time, but a significant number may still experience clinical distress several years after their children complete active treatment (Wijnberg‐Williams, Kamps, Klip, & Hoekstra‐Weebers, 2006). This can negatively impact on family functioning, marital relationships, and has been correlated with self‐reported levels of distress among adolescent cancer survivors (Pai et al., 2007; Trask et al., 2003).

The need for family‐based psychological support was strongly expressed in our study. This is consistent with prior research on childhood cancer in which the family unit is described as the “cancer patient,” and not just the individual diagnosed with cancer (Ljungman et al., 2014). Qualitative studies in USA and Canada have highlighted the potential benefits of retreats and other family‐based interventions to allow survivors, siblings and parents to connect with their own family members and with others in non‐clinical therapeutic settings to build support networks (Bashore & Bender, 2017; Korver, Kinghorn, Negin, Shea‐Perry, & Martiniuk, 2017). Greater family cohesion and support can improve children's adjustment during survivorship, improve their social competence, and reduce the likelihood of posttraumatic stress and behavioural problems (Van Schoors et al., 2017).

The lack of consensus around a single model of care for psychological support was unsurprising, as the severity of psychological distress dictates the level of intervention needed by each individual survivor. Provision of such support should use a stepped care approach to meet individual survivors’ needs and to ensure efficient use of resources (National Cancer Strategy 2017–2026, 2017). Existing research suggests that long‐term health system costs are reduced when survivors experience improved quality of life and fewer psychosocial complications (Carlson & Bultz, 2004).

### 4.1 Strengths

This is the first detailed study of the psychological impact of childhood cancer conducted among adult survivors in Ireland. A multi‐pronged promotion strategy was used to recruit participants for this study, including social media, print media, radio, correspondence with key stakeholders, and email dissemination via the health service and multiple voluntary organisations. This ensured that the study had a wide reach, and it gave participants multiple opportunities to take part.

Open‐ended questions were asked in the focus groups to ensure that complex topics could be discussed, and to identify nuances within group perceptions and beliefs. By asking individuals to write down and/or share their own thoughts at the outset, efforts were made to include all participants in discussions, and to avoid disproportionate focus on the dominant view. A robust analytical process was used, whereby codes, categories, themes and subthemes were audited to enhance trustworthiness of the findings. Member‐check was also used to ensure the results aligned with participants’ views.

### 4.2 Limitations

This was a self‐selected sample, and the majority of participants were female. Those who took part were more likely to be interested and engaged in this area, and may not be representative of the wider population of childhood cancer survivors. We were unable to ask personal information on participants’ diagnoses, age at diagnosis or recurrence. Thus, we were unable to relate individualised demographic or clinical information to any of our findings. Nonetheless, some participants opted to disclose that they had experienced several different diseases and came from at least eight different counties of Ireland, thus they are likely to represent a heterogeneous mix of perspectives. Further research is warranted to identify the psychosocial outcomes among survivors of particular tumour types.

It is plausible that participants who had experienced mental health difficulties may have experienced psychological distress irrespective of any prior cancer diagnosis. It is not possible to establish any causal associations between a previous cancer diagnosis and subsequent distress, but most participants perceived that this was an important contributory factor.

We did not have specific information on the time since survivors had finished their active cancer treatment, thus we cannot exclude the possibility of differential recall among participants in this study. Furthermore, given the exploratory nature of the study, the findings may have been impacted by self‐report bias.

Six of the seven focus groups were facilitated by the same researcher (PMB), a male medical doctor who was a similar age to some of the adult survivors (early 30s). This may have introduced medical preconceptions and assumptions in the research process, or it may have introduced power differences based on sex or education. However, a reflexive approach was taken; a female scribe was present in each of these focus groups, and debriefing was undertaken between the researcher and scribe after each focus group to foster dialogue and to identify any divergent understandings of situations or overlooked perspectives. Furthermore, a female research psychologist (LM) was heavily involved in the analysis and interpretation of the data, and a reflexive dialogue was used to enhance reliability of the findings.

### 4.3 Implications

Since this study was undertaken in April 2018, a number of actions have been progressed. A new National Clinical Lead for Psycho‐Oncology has been appointed to guide the development of psycho‐oncology services in Ireland. This is expected to result in the recruitment of additional qualified psycho‐oncologists to work in the public healthcare system. The findings of this study have been widely communicated to relevant stakeholders including voluntary organisations, paediatric and adult hospitals, and to senior leadership within the health service and the Department of Health to increase awareness of gaps in services, and the existing inequitable access to psychological support.

## 5 CONCLUSION

Adult survivors of childhood cancer and their family members experience enduring psychological effects relating to their diagnosis and treatment. Some survivors and their parents describe delayed trauma several years after completing active cancer treatment, and a minority of both survivors and parents encounter mental health crises. Survivors articulate complex feelings of guilt relating to the perceived burden imposed on their family members during the cancer journey. Parents struggle with fear and guilt relating to their child's ongoing healthcare needs as survivors. Psychological support services are inadequate to meet the needs of this growing population in Ireland. The optimal model of care needs to be flexible to the needs of individuals whose preferences may range from individual counselling to informal group‐based support. Psychological support should be offered to all survivors by default, and should also be available for vulnerable parents and siblings.

## CONFLICT OF INTEREST

The authors declare no conflict of interest.

## AUTHOR CONTRIBUTIONS

PMB and LM contributed to study design, participant recruitment, conduct of focus groups, analysis of transcripts, and drafting and revision of the manuscript. TMcC contributed to study design, interpretation of the findings, drafting and revision of the manuscript. All authors have approved the final version for submission and agree to be accountable for all aspects of the work.
